# West Nile Virus Neuroinvasive Disease: A Retrospective Analysis of Hospitalized Cases in a Tertiary Care Center in Southern Europe

**DOI:** 10.31083/RN36787

**Published:** 2025-09-26

**Authors:** Antonio Cristóbal Luque-Ambrosiani, Ignacio Lopera-Rodríguez, Alicia Fernández-Panadero, María del Sol Torralbo-Gómez, Mikel Salgado-Irazabal, Francisco José Hernández-Chamorro, Francisco José Hernández-Ramos, María Dolores Jiménez-Hernández, Alfredo Palomino-García

**Affiliations:** ^1^Neurology Unit, Virgen del Rocío University Hospital, 41013 Seville, Spain; ^2^Pediatrics Unit, Virgen del Rocío University Hospital, 41013 Seville, Spain; ^3^Neurology Unit, Quirónsalud Sagrado Corazón Hospital, 41013 Seville, Spain

**Keywords:** West Nile virus, critical care, hospital mortality, functional status, prognosis, virus del Nilo Occidental, cuidados intensivos, mortalidad hospitalaria, situación funcional, pronóstico

## Abstract

**Background:**

West Nile virus (WNV) is a flavivirus primarily transmitted by mosquitoes of the *Culex* genus and is endemic to Southern Europe. Although infection is usually asymptomatic, it can lead to neuroinvasive syndromes with high morbidity and mortality. Due to the increasing incidence driven by climatic factors, we present a single-center series examining short- and long-term functional outcomes after infection.

**Methods:**

Patients with neurological symptoms and confirmed WNV infection through serology and/or detection in urine and/or cerebrospinal fluid (CSF) between 2017 and 2023 were included. Data on demographics, medical history, symptoms, diagnostic workup, treatment, and prognosis at discharge, 12 months, and 24 months were analyzed. Patients were categorized based on whether they required intensive care unit (ICU) admission, CSF biochemistry, and treatment employed, among other factors.

**Results:**

Forty patients with a median age of 65 years (45% female) were included; 8% were immunosuppressed. Fever was present in 95%, and 85% experienced prodromal symptoms. Altered consciousness (73%) was the most common neurological symptom. ICU admission was required in 33% of cases, and mechanical ventilation in 25%. In-hospital mortality was 15%. At 24 months, 48% maintained good functional status, with a median follow-up of 35 months. Diagnostic and therapeutic interventions did not influence prognosis.

**Conclusions:**

Although neuroinvasive WNV disease is rare, it carries significant morbidity and mortality, with no specific therapeutic measures impacting outcomes. Prioritizing efforts to control infection spread is critical.

## Abstract (es)

**Introducción:**

El virus del Nilo Occidental es un flavivirus transmitido principalmente por el mosquito del género *Culex* y endémico del sur de Europa. Aunque la infección suele cursar de forma asintomática, puede producir cuadros neuroinvasivos de elevada morbimortalidad. Ante el aumento de incidencia por factores climatológicos, presentamos una serie unicéntrica donde estudiamos situación funcional a corto y largo plazo tras la infección.

**Métodos:**

Se incluyeron pacientes con clínica neurológica y diagnóstico de infección por virus del Nilo Occidental por serología y/o detección en orina y/o líquido cefalorraquídeo entre 2017 y 2023. Se estudiaron características demográficas, antecedentes personales, síntomas, estudio diagnóstico, tratamiento y pronóstico al alta, a los 12 y 24 meses. Se analizaron los pacientes según precisaron o no ingreso en Cuidados intensivos, así como atendiendo a la citobioquímica del líquido cefalorraquídeo y tratamiento empleado, entre otras características.

**Resultados:**

40 pacientes fueron incluidos con mediana de edad de 65 años y 45% de sexo femenino. 8% presentaban inmunosupresión. 95% presentó fiebre y 85% síntomas prodrómicos. La alteración de conciencia (73%) fue el síntoma neurológico más frecuente. 33% requirieron ingreso en Cuidados Intensivos. 25% precisaron ventilación mecánica. 15% fallecieron durante el ingreso. 48% conservaban buena situación funcional a los 24 meses, con mediana de seguimiento de 35 meses. No hubo impacto del resultado del estudio diagnóstico o del tratamiento sobre el pronóstico.

**Conclusiones:**

Pese a la baja frecuencia de enfermedad neuroinvasiva por virus del Nilo Occidental, la morbimortalidad es elevada, sin impacto de medidas terapéuticas específicas, haciendo prioritario el control de propagación de la infección.

## 1. Introduction

West Nile virus (WNV) is the most commonly mosquito-transmitted flavivirus in the world [1]. Humans act as accidental reservoirs within a zoonotic cycle involving birds and horses, where mosquitoes (*Culex spp.*) are the primary vector [2]. In areas such as the Guadalquivir River basin, where migratory birds are prevalent, WNV has become a recurrent public health concern, similar to other regions of Spain [1, 2, 3, 4, 5, 6]. The emergence of milder winters and other environmental changes associated with climate change is increasing vector density and extending their seasonal activity, thereby raising the risk of epidemic outbreaks [7].

Although most infections are asymptomatic or mild, a small percentage (<1%) progress to neuroinvasive disease, such as encephalitis, meningitis, or acute flaccid paralysis, with high morbidity and mortality [8, 9, 10, 11]. This severe progression may be partially mediated by endothelial damage secondary to both systemic and direct inflammation, similar to mechanisms observed in other neuroinfectious diseases [12]. Additionally, the relationship between neuroinfections and cerebrovascular damage has gained attention, as viral infections can exacerbate stroke risk through inflammatory and procoagulant mechanisms, also linked to the impact of climate change on vector-borne diseases [13].

The largest outbreak recorded in Spain occurred in 2020, primarily affecting Andalusia [2], where clinical, neuroimaging, and short-term intrahospital outcomes of neuroinvasive disease were documented [14, 15, 16]. However, there is a lack of studies evaluating the long-term functional impact on patients who survive neuroinvasive disease. This study aims to analyze, through a single-center cohort, the long-term functional outcomes and the relationship between therapeutic measures and prognosis in patients with WNV neuroinfection.

## 2. Materials and Methods

Patients were included through a retrospective analysis of hospital discharge records from Virgen del Rocío University Hospital and outpatient records from hospital specialties (Neurology, Infectious Diseases, and Pediatrics) and Primary Care consultations. Patients were identified retrospectively through hospital records using 10th revision of the International Classification of Diseases (ICD-10) diagnostic codes, including A92.3 (West Nile virus infection) and other related codes for neuroinvasive presentations. In addition, discharge diagnoses were reviewed, and a manual chart review was conducted for all identified cases to confirm that they met the inclusion criteria and fulfilled the case definition for West Nile virus neuroinvasive disease. All patients required hospitalization at this center with a primary diagnosis of WNV neuroinvasive disease (WNND). Hospitalized patients between January 2017 and July 2023 were studied. Data were analyzed in a completely anonymized manner, detached from any information that could identify the patients, and therefore individual written informed consent was not required. Ethical approval was obtained from the local research ethics committee.

Baseline characteristics, including demographic data, medical history, and functional status, were recorded at admission. Functional status at follow-up was assessed using the modified Rankin Scale (mRS). In most cases, the mRS was directly applied during clinical visits by a neurologist. When follow-up was conducted in other departments (e.g., Infectious Diseases, Pediatrics, or Primary Care), and the mRS was not explicitly documented, the score was retrospectively estimated by two authors (ACLA and ILR) based on available clinical records, including the Barthel Index, Lawton–Brody Instrumental Activities of Daily Living (IADL) scale, and qualitative functional assessments.

At 12 months, 8 patients had died and were not available for follow-up. By 24 months, an additional death was recorded, resulting in a total of 9 deceased patients during the study period. No losses to follow-up occurred among the surviving patients, all of whom completed their clinical evaluations at both time points.

For WNV infection, data on the number of hospitalization days, symptoms categorized as prodromal (gastrointestinal, catarrhal, general malaise, and fever), neurological (headache, altered consciousness, ataxia, language disturbance, neck stiffness, paresis, hypoesthesia, vertigo, diplopia, myoclonus, extrapyramidal symptoms, seizures, and delirium), and systemic (skin rash, otalgia, myalgia, and shock) were collected. The use of oxygen therapy, mechanical ventilation, intensive care unit (ICU) admission, and vasoactive therapy with amines was also studied. Additionally, the frequency of in-hospital mortality and other systemic complications were reviewed, as shown in **Supplementary Table 1**.

Cases of WNND were defined following the European Centre for Disease Prevention and Control (ECDC) criteria: Confirmed cases were those with at least one of the following four laboratory tests results: Isolation of WNV from blood or cerebrospinal fluid (CSF); Detection of WNV nucleic acid in blood or CSF; WNV specific antibody response (IgM) in CSF; WNV IgM high titre AND detection of WNV IgG, AND confirmation by neutralisation; Probable cases were those with IgM detected in serum and a clinically compatible presentation, in the absence of an alternative diagnosis.

Clinical syndromes were categorized as West Nile encephalitis, meningitis, or acute flaccid paralysis (AFP). Encephalitis: presence of encephalopathy (e.g., depressed or altered level of consciousness, lethargy, or personality change for ≥24 hours) plus at least two of the following: fever (≥38 °C) or hypothermia (≤35 °C); cerebrospinal fluid pleocytosis (≥5 leukocytes/mm^3^); peripheral leukocyte count >10,000/mm^3^; neuroimaging findings consistent with acute inflammation (with or without involvement of the meninges) or acute demyelination; presence of focal neurologic deficit; meningismus (as defined in A); electroencephalography findings consistent with encephalitis; seizures, either new onset or exacerbation of previously controlled. Meningitis: clinical signs of meningeal inflammation (nuchal rigidity, Kernig/Brudzinski signs, photophobia or phonophobia) plus at least one of the following: fever (≥38 °C) or hypothermia (≤35 °C); cerebrospinal fluid pleocytosis (≥5 leukocytes/mm^3^); peripheral leukocyte count >10,000/mm^3^; or neuroimaging findings consistent with acute meningeal inflammation. Acute flaccid paralysis: acute limb weakness progressing over 48 hours, plus at least two of: asymmetric weakness, areflexia/hyporeflexia, no sensory symptoms, CSF pleocytosis with elevated protein, anterior horn cell findings on electrodiagnostic studies, or spinal cord magnetic resonance imaging (MRI) documenting abnormal increased signal in the anterior gray matter. Cases meeting criteria for encephalitis were classified under that category, even if features of meningitis or AFP were also present.

For the diagnostic study, cerebrospinal fluid (CSF), blood, and urine samples obtained during hospitalization were analyzed. In the CSF, macroscopic appearance, glucose levels, protein levels, leukocytes, polymorphonuclear cells, and qualitative results of polymerase chain reaction (PCR) for WNV and multiplex assays were studied. In blood, WNV-specific immunoglobulins (IgM and IgG) and glucose levels at the time of CSF extraction were analyzed to calculate the glucose consumption index (considered present when the CSF-to-blood glucose ratio was <0.4). In urine, qualitative results of WNV PCR were recorded. Lumbar puncture results, including whether it was traumatic, and findings from neuroimaging (computed tomography [CT] and MRI) and electroencephalogram (EEG) were also reviewed.

For treatment, the use of antivirals, antibiotics, anticonvulsants, and immunomodulators (including corticosteroids and immunoglobulins) was studied. Regarding functional outcomes, mRs scores at discharge, 12 months, and 24 months post-hospitalization were recorded, along with changes in functional status (≥1-point increase), significant functional deterioration (≥2-point increase in mRs), and independence in basic activities of daily living at 24 months. Median follow-up time was also documented.

To evaluate potential predictors of long-term functional outcome, patients were stratified according to their mRS score at 12 and 24 months. A “good outcome” was defined as an mRS score of 0–2, indicating functional independence in activities of daily living. Comparative analysis was performed between patients with good vs. poor outcomes, examining variables including demographic characteristics, vascular risk factors, toxic habits (e.g., smoking, alcohol use), CSF findings, clinical presentation subtype (encephalitis, meningitis, acute flaccid paralysis), and treatments received. Due to sample size limitations, only bivariate comparisons were conducted.

The statistical analysis began with a descriptive evaluation of the included variables according to their type. For quantitative variables, distributions were assessed for normality using the Shapiro-Wilk test. Quantitative variables were reported as medians and interquartile ranges (IQRs) due to the non-normal distribution of some variables. Qualitative variables were expressed as percentages. Results were presented in rounded whole numbers, except for hypothesis testing, where significance was reported to three decimal places. Comparisons of qualitative variables were performed using the χ^2^ test or Fisher’s exact test. Quantitative variables were compared using the Student’s *t*-test or the Mann-Whitney *U* test, depending on the normality of the distribution. Statistical significance was set at *p* < 0.05. All statistical analyses were performed using SPSS v29 (IBM, Armonk, NY, USA).

## 3. Results

Between January 2017 and August 2023, 40 patients were included based on the inclusion criteria of hospital admission with a primary diagnosis of WNND. Regarding demographic characteristics, the median age was 65 years (IQR 37–77 years), and 45% of the patients were female. A total of 25% were smokers, 1 individual had a history of alcoholism, and no patients reported drug use. Hypertension was present in 45%, dyslipidemia in 25%, diabetes in 23%, stroke in 13%, ischemic heart disease in 10%, chronic obstructive pulmonary disease in 8%, chronic kidney disease in 8%, immunosuppression in 8%, heart failure in 5%, obesity in 2 individuals, and intermittent claudication in 1 individual. A total of 95% of patients had good baseline functional status, with an mRs ≤2.

**Fig. 1.**
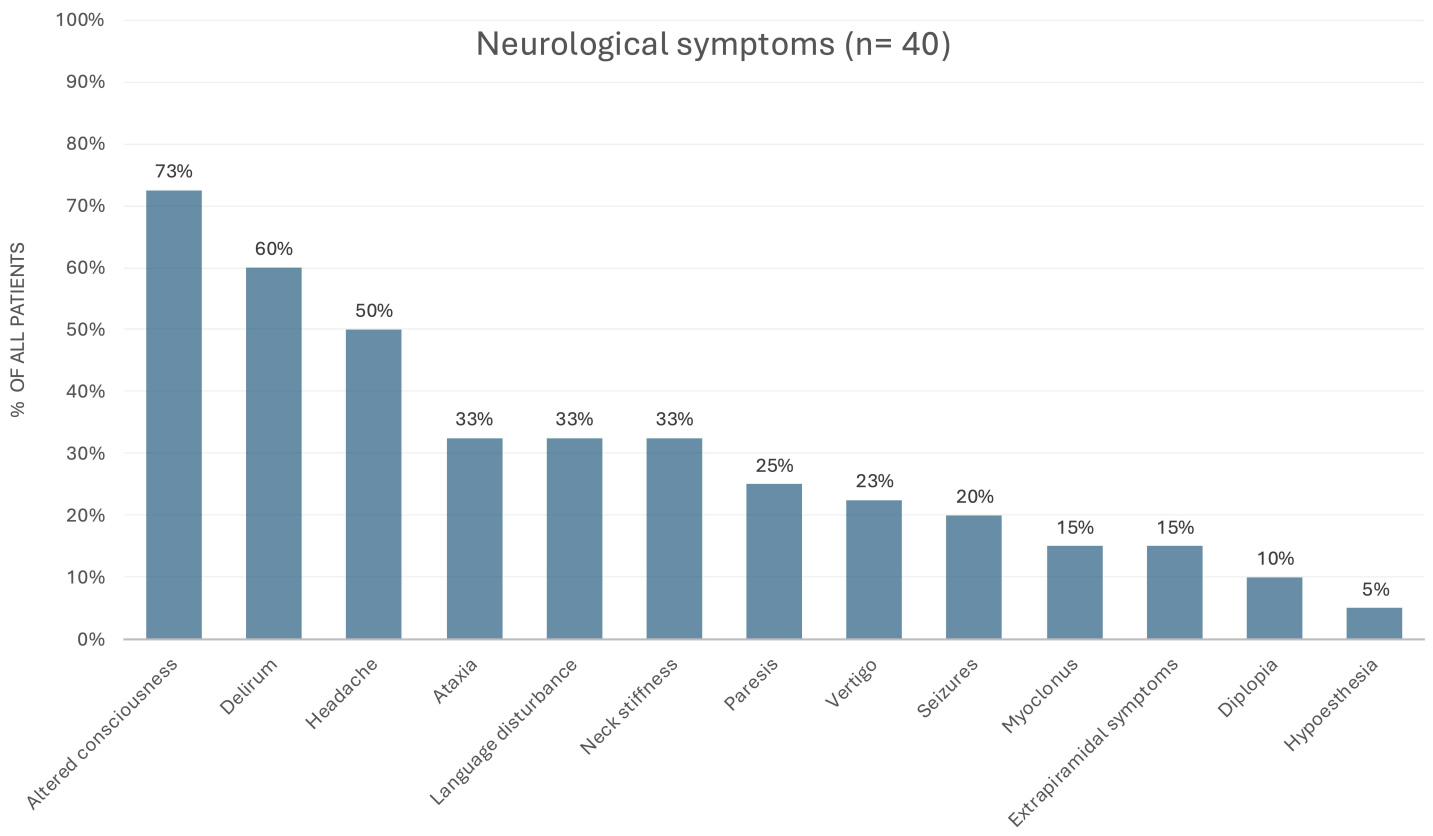
**Description of neurological symptoms, ordered by frequency of occurrence**.

In terms of symptoms, 95% of patients presented with fever, and 85% reported prodromal symptoms, which preceded neurological involvement as detailed in Fig. 1. Prodromal symptoms were documented in 34 of the 40 patients. Beyond fever and headache (analyzed separately), the most frequently reported symptoms included fatigue or malaise, nausea, vomiting and arthralgia. The most common neurological symptoms were altered consciousness (73%), delirium (60%), headache (50%), ataxia (33%), language disturbance (33%), and neck stiffness (33%). Additionally, 33% experienced myalgia, 15% had a skin rash, and 10% reported otalgia. 24 (60%) were classified as having encephalitis, 10 (25%) as meningitis, and 6 (15%) as acute flaccid paralysis based on clinical criteria. Seizures were observed in 8 patients, all diagnosed based on clinical assessment, either through direct observation during hospitalization or via anamnesis. No electrographic seizures were identified in the EEGs performed, and no cases of status epilepticus were documented. The median hospital stay was 10 days (IQR 5–17 days). Oxygen therapy was required in 48%, mechanical ventilation in 25%, and vasoactive amines were administered in 13%. ICU admission was necessary for 33% of patients. Shock (defined as arterial hypotension refractory to fluid resuscitation, including vasoactive amines) occurred in 13%. Systemic complications were observed in 45% of patients. In-hospital mortality was 15%.

Regarding the diagnostic study, serology and PCR for WNV in urine were performed in all cases, and PCR for WNV in CSF was conducted in 98%. The serological profile at diagnosis consisted of 65% IgM+ and IgG–, 30% IgM+ and IgG+, 1 patient with IgM– and IgG+, and 1 patient with IgM– and IgG–. PCR for WNV in urine was positive in 13% of cases, and a similar result was found in 13% for PCR in CSF. 19 cases (48%) met criteria for confirmed WNND, and 21 cases (53%) were classified as probable (**Supplementary Table 2**). For the CSF analysis, the cytobiochemical results are summarized in Table 1. The median total protein (62 mg/dL) and leukocyte count (55 cells/mm^3^) were within pathological ranges (>50 mg/dL and >5 cells/mm^3^, respectively), with a clear predominance of monocytes (median of 35% polymorphonuclear cells). Of the 39 patients with available CSF cytology, 17 (38%) showed a predominance of polymorphonuclear cells (>50% of total leukocytes). This neutrophilic pattern was observed across different clinical presentations, including encephalitis and meningitis. A total of 95% of lumbar punctures were atraumatic, 90% of CSF samples had a clear appearance, and only 5% showed glucose consumption. Quantitative paired CSF-serum immunoglobulin measurements were not routinely available, and therefore a full Reiber diagram analysis could not be performed in our cohort.

**Table 1.** **Overall profile of CSF cytobiochemical results (n = 39)**.

CSF cytobiochemistry	Median	IQR	P25	P75
Glucose (mg/dL)	68	32	58	90
Total proteins (mg/dL)	62	50	43	93
Leukocytes (cells/mm^3^)	55	146	17	163
Polymorphonuclear cells (% of total)	35	52	16	68
Red blood cells (cells/mm^3^)	0	3	0	3

CSF, cerebrospinal fluid; IQR, interquartile range; P25, 25th percentile; P75, 75th percentile.

Regarding neuroimaging studies, all patients underwent urgent cranial CT scans, which were normal in 98% of cases. Cranial MRI was performed during hospitalization in 60% of patients, and it was pathological in 25% of the total sample. The most frequent finding was T2 hyperintensity in the brainstem and thalamus. Patients with pathological findings on brain MRI showed higher mRS scores at both 12 and 24 months of follow-up (medians 4.5 and 5.0, respectively), compared to those with normal imaging (median 2.0 in both cases). However, these differences did not reach statistical significance (*p* = 0.202 and *p* = 0.312, respectively; Mann–Whitney U test). EEGs were conducted in 48% of patients, with the most common result being normal (18%), followed by mild generalized dysfunction (13%) and moderate dysfunction (10%). Only one patient showed epileptiform abnormalities, but without features consistent with electrographic seizures or status epilepticus.

In terms of treatment, 63% of patients received antiviral therapy (mainly acyclovir), 65% received antibiotics, and 33% were treated with anticonvulsants (8 patients were treated therapeutically, following the occurrence of clinical seizures; the remaining 5 patients received AEDs prophylactically, most commonly in the setting of ICU admission without access to continuous EEG monitoring, based on clinical judgment to avoid missing potential subclinical seizures). Acyclovir was initiated in all patients at the time of suspected central nervous system infection, and was generally maintained until Herpes Simplex Virus (HSV) encephalitis could be reasonably excluded. In most cases, this corresponded to the timing of a second negative lumbar puncture or confirmation of an alternative diagnosis (WNV). Although individual treatment durations were not consistently documented, the majority of patients received either a complete course or a substantial portion of antiviral therapy. A total of 30% did not receive immunomodulators; among those who did, 53% were treated exclusively with corticosteroids, and 18% received both corticosteroids and immunoglobulins.

**Fig. 2.**
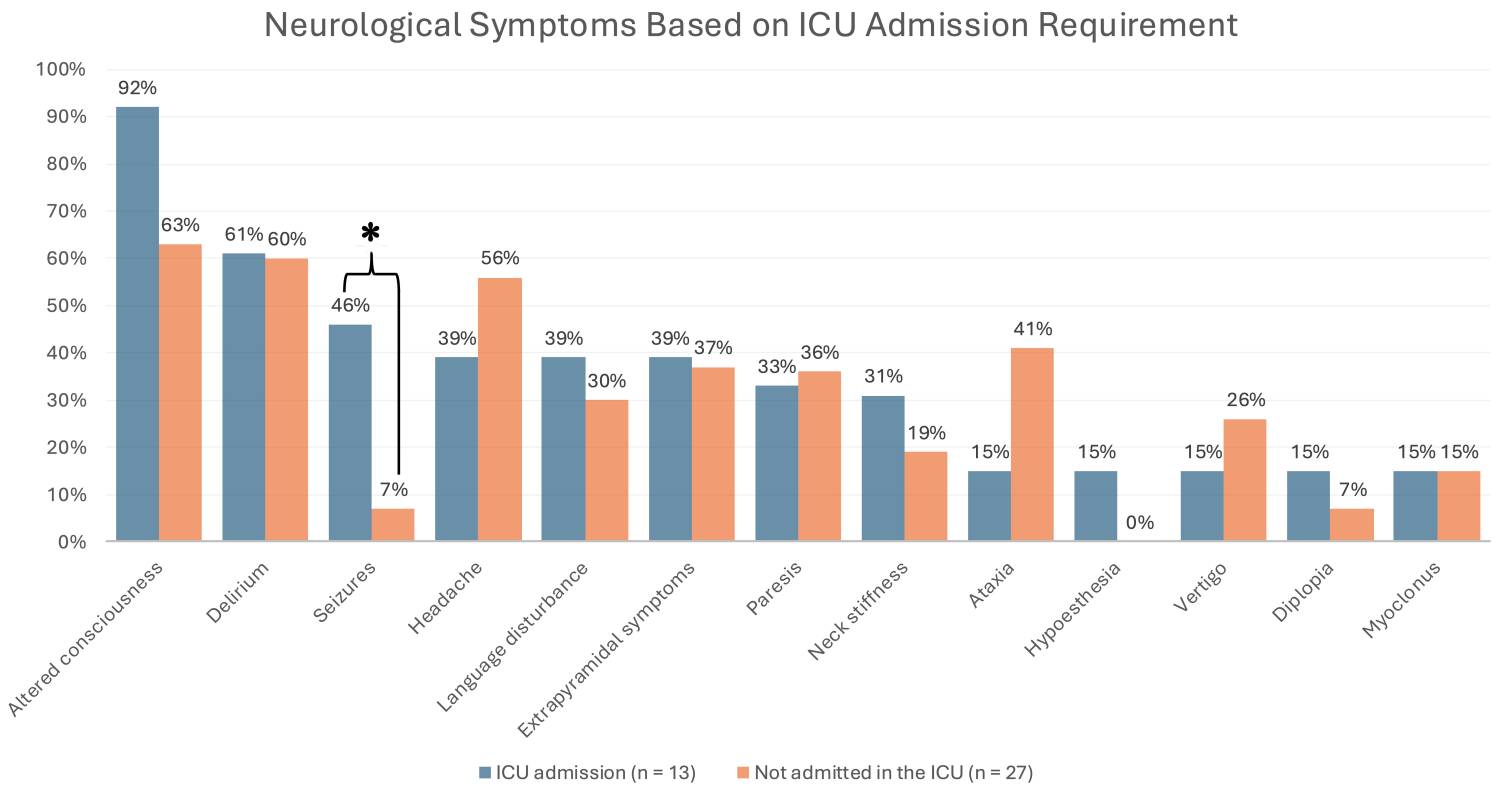
**Description of neurological symptoms based on whether intensive care unit (ICU) admission was required or not (n = 40)**. *, *p* < 0.05.

Alongside the analysis of the overall sample, special attention was given to patients based on whether they required ICU admission or not. Among those admitted to the ICU, the three most common neurological symptoms were altered consciousness (92%), delirium (61%), and seizures (46%). The latter showed a statistically significant difference compared to patients who did not require ICU admission (46% vs. 7%, *p* = 0.008), as shown in Fig. 2.

Regarding demographic characteristics, personal history, and other symptoms, no statistically significant differences were observed in most parameters between groups, except for a longer hospital stay in patients who required ICU admission (17 vs. 7 days; *p* < 0.001). Similarly, no statistically significant differences were found in CSF cytobiochemistry. However, significant differences were observed in the following aspects: higher prevalence of shock (31% vs. 4%; *p* = 0.031), need for oxygen therapy (92% vs. 26%; *p* < 0.001), mechanical ventilation (62% vs. 7%; *p* < 0.001), use of vasoactive amines (31% vs. 4%; *p* = 0.031), systemic complications (77% vs. 30%; *p* = 0.007), less frequent use of corticosteroids alone (46% vs. 56%; *p* = 0.002), and greater use of corticosteroids combined with immunoglobulins (46% vs. 4%; *p* = 0.002). No statistically significant differences were observed in other variables, including neuroimaging, EEG findings, functional status over the follow-up period, or WNV diagnostic profiles in the various samples analyzed.

**Fig. 3.**
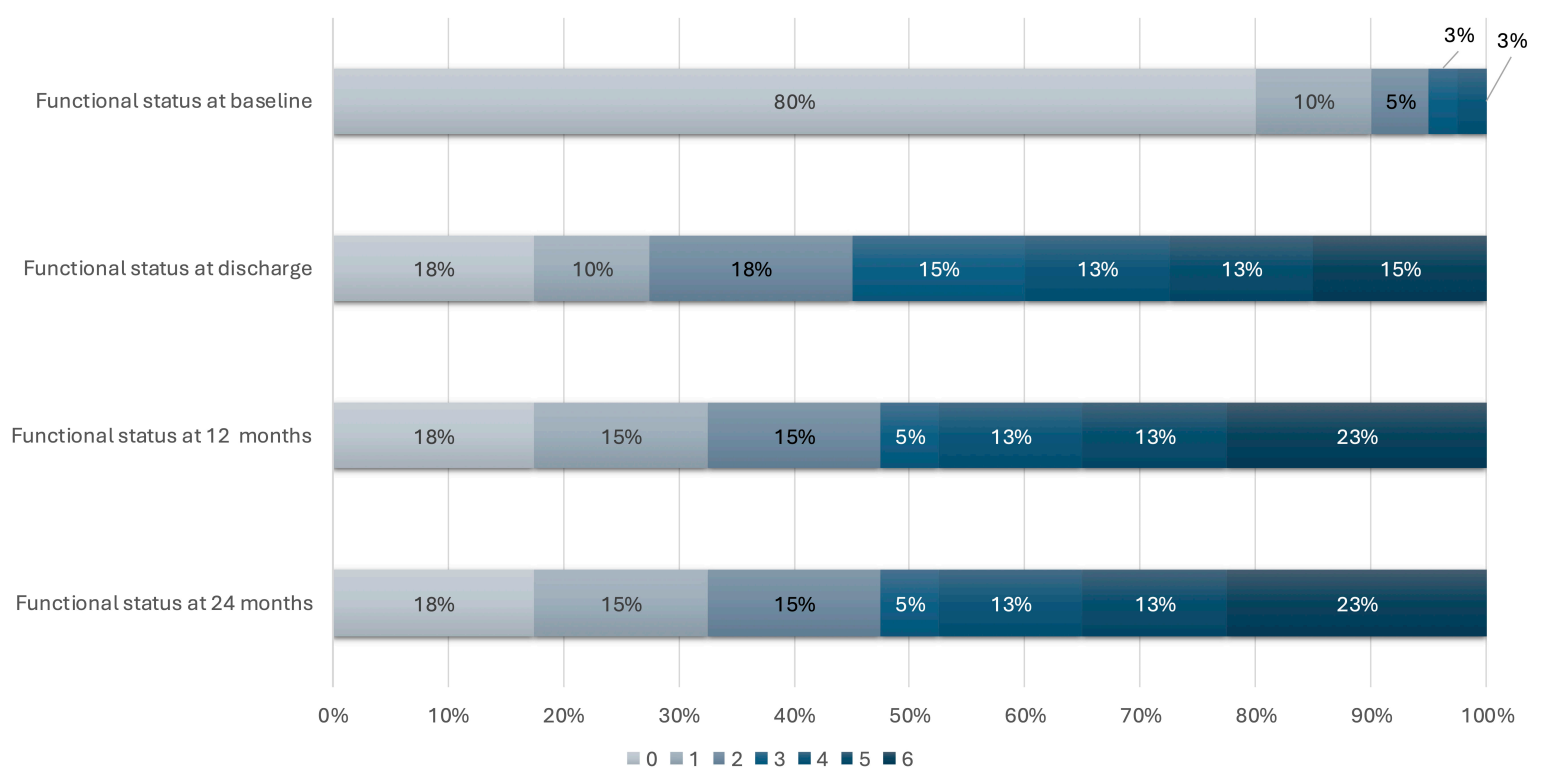
**Functional status over the follow-up period (n = 40), shown according to modified Rankin Scale (mRS) scores (0–6)**.

Regarding functional status, results are shown in Fig. 3. A total of 95% of patients had good baseline functional status. However, at discharge (46%), 12 months (48%) and 24 months (48%), there was a decline of over 40% in the proportion of patients with good functional status compared to baseline. A significant change in functional deterioration (≥2-point increase in mRs when comparing baseline to 24 months) was observed in 42.5% of patients. A moderate, statistically significant positive correlation was observed between age and functional disability at both 12 months (Spearman’s ρ = 0.475; *p* = 0.002) and 24 months (ρ = 0.549; *p* < 0.001). This suggests that older age was associated with worse long-term outcomes. The strength of this association increased slightly at 24 months. The 95% confidence intervals were 0.182–0.690 and 0.277–0.739, respectively. The median follow-up was 35 months (IQR 35–36), with most cases concentrated in the summer of 2020, primarily in the areas of La Puebla and Coria del Río.

No statistically significant differences were found between the presence of pathological CSF findings (defined as elevated protein and/or cell counts above the normal range per the local laboratory reference) or treatment and functional status at different follow-up time points. Similarly, no differences were observed in mortality rates or functional outcomes according to the clinical syndrome subtype (encephalitis, meningitis, or acute flaccid paralysis). When comparing patients by long-term functional outcome (mRS ≤2 vs. >2), no statistically significant associations were found with baseline characteristics, vascular risk factors, CSF profiles, clinical presentation, or treatments administered. These findings likely reflect the limited statistical power of the study and should be interpreted with caution.

## 4. Discussion

In this study, we report 40 cases of patients with neuroinvasive WNV disease in an endemic regional context. The findings highlight the considerable clinical and functional impact of this condition, characterized by high mortality (15% in-hospital and 23% at 24 months) and significant functional deterioration in nearly half of the patients after two years of follow-up.

Regarding demographic characteristics, the median age of 65 years observed in our cohort is consistent with previous reports of WNV neuroinvasive disease primarily affecting older adults [9, 10, 11]. Only 8% of patients were immunosuppressed, a condition that has been associated with poorer prognosis in an earlier study [17]; however, no clear relationship with worse outcomes was found in our sample. Encephalitis emerged as the most frequent clinical presentation (73%), followed by meningitis (33%) and acute flaccid paralysis (25%), with some degree of clinical overlap. While we observed no significant differences in baseline characteristics, mortality, or long-term functional outcomes across clinical subtypes, these comparisons must be interpreted with caution given the limited statistical power of our sample. Nonetheless, the distribution of syndromes and their outcomes appear in line with other published series, reinforcing encephalitis as the most frequent neuroinvasive form of WNV infection. This clinical pattern aligns with previous studies that underscore the association between encephalitis, endothelial inflammation, and blood–brain barrier disruption—mechanisms widely described in neuroinvasive flavivirus infections such as West Nile virus. Although no cerebrovascular events were documented in our cohort, we retained this discussion due to increasing evidence suggesting that viral encephalitis may promote vascular endothelial damage and a prothrombotic milieu, potentially predisposing to delayed stroke and other complications. These processes, supported by both clinical and experimental data, are not unique to WNV but have been observed across various neurotropic flaviviruses. Our aim is not to extrapolate beyond the data, but rather to situate our findings—particularly the predominance of encephalitis—within the broader pathophysiological context. This perspective is consistent with emerging discussions in the literature and serves a hypothesis-generating purpose, highlighting the need for future research into cerebrovascular outcomes following WNND [7, 12, 13].

Compared to published data, our findings are similar to a smaller series from the same province, which reported 63% of cases as encephalitis [16]. However, they differ from other regions where meningitis was the most frequent manifestation [9, 11]. Additionally, 33% of our sample required ICU admission, and 25% needed mechanical ventilation. Mortality in our series was higher than reported in other studies, with 15% in-hospital mortality and 23% at 24 months, compared to approximately 10% in previous reports [9, 10]. We found no differences in mortality based on clinical manifestations, a finding also consistent with the literature [9].

The diagnosis of WNND relies on a multimodal approach that integrates clinical features with serologic and molecular testing. In our cohort, diagnosis was primarily based on the detection of IgM and IgG in serum and CSF, as well as PCR testing, mainly in CSF or urine. While CSF IgM is considered confirmatory, its persistence over time may lead to diagnostic ambiguity, and serum IgM is susceptible to false positives due to flavivirus cross-reactivity [9, 10, 11]. PCR testing, although less frequently positive, was diagnostic in 13% of our patients and proves especially useful in early-phase infection or in immunocompromised individuals [10]. These findings highlight the importance of combining different diagnostic tools to optimize accuracy and reduce misclassification [9, 10, 11].

Regarding complementary studies, CSF analysis showed an inflammatory profile with lymphocytic predominance, elevated protein levels, and no glucose consumption in most cases, consistent with expectations. A predominance of neutrophils in the CSF, observed in 38% of our cases, has been previously reported in WNND and other flavivirus infections such as tick-borne encephalitis. This finding, although atypical for viral meningitis or encephalitis in general, may serve as an early diagnostic clue suggestive of WNND, particularly in the appropriate epidemiological context (Pelz *et al*., 2024 [18]; Senel *et al*., 2020 [19]). Our results support its inclusion in the differential diagnostic reasoning for Central Nervous System (CNS) infections in endemic areas. Although the Reiber diagram has been proposed as a useful tool to detect early intrathecal IgM synthesis in cases of West Nile neuroinvasive [18, 19], this analysis could not be performed in our cohort, as paired quantitative measurements of immunoglobulins in CSF and serum were not systematically available. In terms of neuroimaging, while findings in other series vary, diencephalic (mainly thalamic) and brainstem involvement predominated in our sample. Although the differences in functional outcome did not reach statistical significance, the observed trend is clinically relevant: patients with pathological brain MRI findings, particularly those with lesions in deep structures such as the thalamus or brainstem, tended to have poorer long-term recovery. This pattern has been reported in other cohorts and suggests that structural CNS involvement may be associated with sustained disability [9, 10, 11, 16, 17].

The functional impact of WNND is particularly noteworthy, as nearly half of the patients in our cohort experienced substantial deterioration in their quality of life, transitioning from full independence to requiring assistance with daily activities or even continuous care. This observation is consistent with previous studies reporting complete recovery rates below 40% at one year of follow-up [9, 10]. In our cohort, functional impairment persisted up to 24 months, with a median follow-up of over two and a half years, reinforcing the need to consider WNND as a condition with potential for chronic disability. Furthermore, our data suggest that increasing age is associated with worse long-term recovery, a trend also supported by prior research in neuroinfectious diseases, including WNV.

While recovery trajectories vary, only a subset of patients achieve full functional recovery, and many continue to experience persistent neurological deficits months or even years after the acute illness. The Houston West Nile virus cohort offers some of the most robust prospective evidence on this topic, showing that neurological impairment remained in nearly half of patients one year post-infection, particularly among those with encephalitis or acute flaccid paralysis. Notably, acute flaccid paralysis is consistently associated with the worst long-term outcomes, due to irreversible anterior horn cell injury and motor neuron loss. Similarly, encephalitis is often followed by residual cognitive and motor deficits, whereas meningitis is typically associated with more favorable outcomes, despite the possibility of lingering fatigue and neurocognitive symptoms [20]. These findings reinforce the importance of early neurorehabilitation, structured long-term follow-up, and heightened clinical awareness of the chronic impact of WNND on survivors.

Therapeutic management remains primarily supportive, as no significant long-term prognostic impact has been demonstrated for antiviral or immunomodulatory treatments used [11]. Patients who required ICU admission showed a higher prevalence of the encephalitic phenotype (92%), longer hospital stays, greater systemic severity, and a higher need for invasive support. However, we found no association between functional outcomes and CSF cytobiochemistry, which does not support its utility as a prognostic factor.

These findings underscore the importance of implementing more effective preventive strategies, such as vector control and epidemiological surveillance, particularly in the context of climate change and its impact on the spread of vector-borne diseases [4]. Furthermore, the observed functional deterioration reinforces the need to allocate resources to rehabilitation and care for dependent patients, considering that nearly 50% of patients in our series did not regain their baseline functionality after 24 months of follow-up [5, 11].

Finally, the link between climate change, endothelial damage, and the increasing burden of stroke associated with viral infections like WNV should be a key focus of future research [7, 12, 13]. Understanding these mechanisms could enable the development of comprehensive interventions to mitigate the long-term impact of these diseases. Limitations of this study include its retrospective nature and the small sample size for subgroup analyses, which may limit the ability to identify statistically significant differences.

## 5. Conclusions

Neuroinvasive WNV disease, although rare, has a significant impact on the functional status of patients, as well as high mortality. It is crucial to allocate more resources to preventive measures to avoid epidemic outbreaks, given the lack of demonstrated efficacy of current treatments and the potential persistence of other morbid factors that may explain the increase in long-term disability and delayed mortality. Alongside primary prevention as a fundamental pillar, directing resources toward rehabilitation and care for dependent patients is essential for those who experience greater deterioration in their functional status, due to its substantial impact.

## Data Availability

The datasets used and analyzed during the current study are available from the corresponding author on reasonable request.
