# Supplementary material for: West Nile Virus Neuroinvasive Disease: A Retrospective Analysis of Hospitalized Cases in a Tertiary Care Center in Southern Europe
Source: Rev Neurol. 2025 Sep 26;80(8):36787. doi: 10.31083/RN36787 (PMC12516826; doi:10.31083/RN36787)
Supplement: Translation

# Translation: Enfermedad Neuroinvasiva por Virus del Nilo Occidental: Análisis Retrospectivo de Casos Hospitalizados en un Centro de Tercer Nivel del Sur de Europa

**Published:** 2025-09-26

**Authors:** Antonio Cristóbal Luque-Ambrosiani, Ignacio Lopera-Rodríguez, Alicia Fernández-Panadero, María del Sol Torralbo-Gómez, Mikel Salgado-Irazabal, Francisco José Hernández-Chamorro, Francisco José Hernández-Ramos, María Dolores Jiménez-Hernández, Alfredo Palomino-García

**Affiliations:** ^4^Unidad de Gestión Clínica de Neurología, Hospital Universitario Virgen del Rocío, 41013 Sevilla, España; ^5^Unidad de Gestión Clínica de Pediatría, Hospital Universitario Virgen del Rocío, 41013 Sevilla, España; ^6^Servicio de Neurología, Hospital Quirónsalud Sagrado Corazón, 41013 Sevilla, España

**Keywords:** virus del Nilo Occidental, cuidados intensivos, mortalidad hospitalaria, situación funcional, pronóstico

## Abstract

**Introducción:**

El virus del Nilo Occidental es un flavivirus transmitido principalmente por el mosquito del género *Culex* y endémico del sur de Europa. Aunque la infección suele cursar de forma asintomática, puede producir cuadros neuroinvasivos de elevada morbimortalidad. Ante el aumento de incidencia por factores climatológicos, presentamos una serie unicéntrica donde estudiamos situación funcional a corto y largo plazo tras la infección.

**Métodos:**

Se incluyeron pacientes con clínica neurológica y diagnóstico de infección por virus del Nilo Occidental por serología y/o detección en orina y/o líquido cefalorraquídeo entre 2017 y 2023. Se estudiaron características demográficas, antecedentes personales, síntomas, estudio diagnóstico, tratamiento y pronóstico al alta, a los 12 y 24 meses. Se analizaron los pacientes según precisaron o no ingreso en Cuidados intensivos, así como atendiendo a la citobioquímica del líquido cefalorraquídeo y tratamiento empleado, entre otras características.

**Resultados:**

40 pacientes fueron incluidos con mediana de edad de 65 años y 45% de sexo femenino. 8% presentaban inmunosupresión. 95% presentó fiebre y 85% síntomas prodrómicos. La alteración de conciencia (73%) fue el síntoma neurológico más frecuente. 33% requirieron ingreso en Cuidados Intensivos. 25% precisaron ventilación mecánica. 15% fallecieron durante el ingreso. 48% conservaban buena situación funcional a los 24 meses, con mediana de seguimiento de 35 meses. No hubo impacto del resultado del estudio diagnóstico o del tratamiento sobre el pronóstico.

**Conclusiones:**

Pese a la baja frecuencia de enfermedad neuroinvasiva por virus del Nilo Occidental, la morbimortalidad es elevada, sin impacto de medidas terapéuticas específicas, haciendo prioritario el control de propagación de la infección.

## 1. Introducción

El virus del Nilo Occidental (VNO) es el flavivirus más comúnmente transmitido por mosquitos en el mundo [1]. Los seres humanos actúan como reservorios accidentales dentro de un ciclo zoonótico entre aves y caballos, donde el mosquito (*Culex* spp.) es el principal vector [2]. En áreas como la cuenca del Guadalquivir, donde las aves migratorias son frecuentes, el VNO se ha convertido en un problema de salud pública recurrente, de forma similar a otras regiones de España [1, 2, 3, 4, 5, 6]. La aparición de inviernos más cálidos y otros cambios ambientales asociados al cambio climático están aumentando la densidad de vectores y prolongando su actividad estacional, lo que incrementa el riesgo de brotes epidémicos [7].

Aunque la mayoría de las infecciones son asintomáticas o leves, un pequeño porcentaje (<1%) evoluciona hacia enfermedad neuroinvasiva, como encefalitis, meningitis y parálisis flácida aguda, con alta morbimortalidad [8, 9, 10, 11]. Esta progresión grave puede estar mediada, en parte, por daño endotelial secundario a inflamación sistémica y directa, similar a mecanismos observados en otras enfermedades neuroinfecciosas [12]. Además, la relación entre neuroinfecciones y el daño cerebrovascular ha cobrado relevancia, dado que infecciones virales pueden exacerbar el riesgo de ictus a través de mecanismos inflamatorios y procoagulantes, vinculados también al impacto del cambio climático en enfermedades vectoriales [13].

El brote más grande registrado en España tuvo lugar en 2020, centrado en Andalucía [2], del que recogieron datos clínicos, de neuroimagen y sobre evolución intrahospitalaria de enfermedad neuroinvasiva a corto plazo [14, 15, 16]. Sin embargo, se carece de estudios que evalúen el impacto funcional a largo plazo en pacientes que sobreviven a la enfermedad neuroinvasiva. Este estudio tiene como objetivo analizar, mediante una cohorte unicéntrica, los desenlaces funcionales a largo plazo y la relación entre las medidas terapéuticas y el pronóstico en pacientes con neuroinfección por VNO.

## 2. Materiales y Métodos

Se incluyeron, mediante análisis retrospectivo de Altas Hospitalarias del Hospital Universitario Virgen del Rocío y episodios de Consulta en Atención Hospitalaria (Neurología, Enfermedades Infecciosas y Pediatría) y Atención Primaria, pacientes que requirieron ingreso hospitalario en dicho centro con diagnóstico principal de enfermedad neuroinvasiva por virus del Nilo Occidental. Los pacientes fueron identificados de forma retrospectiva a partir de los registros hospitalarios mediante el uso de códigos diagnósticos Clasificación internacional de enfermedades décima edición (CIE-10), incluyendo A92.3 (infección por virus del Nilo Occidental) y otros códigos relacionados con presentaciones neuroinvasivas. Además, se revisaron los diagnósticos al alta y se realizó una revisión manual de las historias clínicas para confirmar que los casos cumplían con los criterios de inclusión y la definición de caso de enfermedad neuroinvasiva por virus del Nilo Occidental. Se estudiaron pacientes hospitalizados entre enero de 2017 y julio de 2023. Se analizaron los datos desligados de cualquier información que pudiese permitir su identificación, realizando su estudio de forma completamente anonimizada, por lo que no fue necesario recabar consentimiento informado por escrito de forma individual. Sí se contó con aprobación del comité de ética de la investigación local.

Se registraron las características basales, incluyendo datos demográficos, antecedentes médicos y situación funcional al ingreso. El estado funcional en el seguimiento se evaluó mediante la escala de Rankin modificada (mRS). En la mayoría de los casos, la escala fue aplicada directamente por un neurólogo durante las visitas clínicas. Cuando el seguimiento tuvo lugar en otros servicios (como Enfermedades Infecciosas, Pediatría o Atención Primaria), y no se documentó explícitamente la mRS, esta fue estimada retrospectivamente por dos autores (ACLA e ILR) a partir de la información clínica disponible, incluyendo el Índice de Barthel, la escala de Lawton–Brody y descripciones funcionales en la historia clínica.

A los 12 meses, 8 pacientes habían fallecido, por lo que no se realizó seguimiento de la situación funcional en esos casos. A los 24 meses, se registró un fallecimiento adicional, con un total de 9 muertes durante el periodo de estudio. No se registraron pérdidas al seguimiento entre los pacientes supervivientes, quienes completaron las evaluaciones clínicas en ambos momentos.

En cuanto a la infección por VNO, se registraron días de hospitalización, síntomas clasificados como prodrómicos (gastrointestinales, catarrales, malestar general y fiebre) y aquellos que se encuadraron como neurológicos (cefalea, alteración de conciencia, ataxia, alteración del lenguaje, rigidez nucal, paresia, hipoestesia, vértigo, diplopía, mioclonías, síntomas extrapiramidales, crisis comicial y síndrome confusional), así como sistémicos (exantema cutáneo, otalgia, mialgias y shock). También se estudiaron uso de oxigenoterapia, necesidad de ventilación mecánica, ingreso en Unidad de Cuidados Intensivos (UCI) y terapia vasoactiva con aminas. Asimismo, se revisó la frecuencia de muerte intrahospitalaria y aparición de otras complicaciones sistémicas, mostradas en el **Tabla 1 del Material Suplementario**.

Los casos de enfermedad neuroinvasiva por virus del Nilo Occidental se definieron de acuerdo con los criterios del European Centre for Disease Prevention and Control (ECDC). Se consideraron casos confirmados aquellos con al menos uno de los siguientes resultados de laboratorio: Aislamiento del virus del Nilo Occidental (VNO) en sangre o líquido cefalorraquídeo (LCR); Detección de ácido nucleico de WNV en sangre o LCR; Respuesta de anticuerpos específicos IgM frente a WNV en LCR; Título alto de IgM frente a WNV junto con detección de IgG y confirmación mediante neutralización. Se consideraron casos probables aquellos con IgM frente a WNV detectada en suero, una presentación clínica compatible y sin diagnóstico alternativo.

Los síndromes clínicos se clasificaron como encefalitis por virus del Nilo Occidental, meningitis o parálisis flácida aguda (PFA). Encefalitis: presencia de encefalopatía (alteración del nivel de conciencia, letargia o cambios de personalidad ≥24 horas), más al menos dos de los siguientes criterios: Fiebre (≥38 °C) o hipotermia (≤35 °C); Pleocitosis en LCR (≥5 leucocitos/mm^3^); Leucocitosis en sangre >10.000/mm^3^; Hallazgos en neuroimagen compatibles con inflamación aguda (con o sin afectación meníngea) o desmielinización aguda; Déficit neurológico focal; Meningismo; Hallazgos en EEG compatibles con encefalitis; Crisis epilépticas de nueva aparición o exacerbación de crisis previamente controlada. Meningitis: signos clínicos de inflamación meníngea (rigidez de nuca, signos de Kernig/Brudzinski, fotofobia o fonofobia) más al menos uno de los siguientes: Fiebre (≥38 °C) o hipotermia (≤35 °C); Pleocitosis en LCR (≥5 leucocitos/mm^3^); Leucocitosis en sangre >10.000/mm^3^; Hallazgos en neuroimagen compatibles con inflamación meníngea aguda. PFA: debilidad aguda de las extremidades con progresión durante 48 horas, más al menos dos de los siguientes: Debilidad asimétrica; Arreflexia o hiporreflexia; Ausencia de síntomas sensitivos; Pleocitosis en LCR con hiperproteinorraquia; Hallazgos en estudios electrofisiológicos compatibles con afectación de la motoneurona anterior; resonancia magnética (RM) medular con aumento de señal en la sustancia gris anterior. Los casos que cumplían criterios de encefalitis se clasificaron bajo dicha categoría, incluso si presentaban también signos de meningitis o PFA.

Respecto al estudio diagnóstico, se analizaron muestras de LCR, sangre y orina obtenidas durante la hospitalización, estudiándose en el LCR: aspecto macroscópico, glucosa, proteinorraquia, leucocitos, polimorfonucleares, resultado cualitativo de la reacción en cadena de la polimerasa (PCR) de VNO y multiplex; en sangre: inmunoglobulinas (Ig) M y G frente VNO y glucemia al extraer LCR para calcular consumo de glucorraquia (presente cuando el resultado de dividir glucorraquia entre glucemia era menor que 0,4). En orina: resultado cualitativo de PCR de VNO. Se registró si la punción lumbar fue traumática, así como resultados de neuroimagen (incluyendo tomografía computarizada (TC) y RM) y electroencefalograma (EEG).

Acerca del tratamiento, se estudiaron uso de antivirales, antibióticos, anticomiciales e inmunomoduladores (incluyendo corticoesteroides e inmunoglobulinas). Respecto al resultado funcional, se registró situación funcional medida por mRs al alta, a los 12 y 24 meses del ingreso, así como cambio en la situación funcional (incremento mayor o igual a 1 punto), empeoramiento en situación funcional significativo (aumento mayor o igual 2 puntos en escala mRs) e independencia al menos para actividades básicas de la vida diaria a los 24 meses, incluyéndose mediana de seguimiento.

Para evaluar posibles predictores del desenlace funcional a largo plazo, los pacientes fueron clasificados según su puntuación en la escala mRS a los 12 y 24 meses de seguimiento. Se definió como “buen desenlace” una puntuación de 0 a 2 en la mRS, lo que indica independencia funcional para las actividades de la vida diaria. Se realizó un análisis comparativo entre los grupos con buen y mal desenlace, considerando variables como las características demográficas, factores de riesgo vascular, hábitos tóxicos (p. ej., tabaquismo, consumo de alcohol), parámetros del líquido cefalorraquídeo, tipo de presentación clínica (encefalitis, meningitis, parálisis flácida aguda) y tratamientos recibidos. Debido al tamaño muestral, solo se llevaron a cabo comparaciones bivariantes.

El análisis estadístico consistió, en primer lugar, en la valoración descriptiva de las variables incluidas, según su tipología. En las cuantitativas se estudió su distribución para conocer si cumplían la hipótesis de normalidad mediante test de Shapiro-Wilk. Se reportaron las variables cuantitativas mediante mediana y rango intercuartílico, ya que algunas no seguían distribución normal. Las cualitativas se expresaron mediante porcentajes. Se presentaron resultados en números enteros con redondeo hacia la unidad más cercana, a excepción del cálculo de hipótesis donde se reportó significación incluyendo hasta milésima. Se realizó la comparación de variables cualitativas mediante test χ^2^ o test exacto de Fisher. La comparación de variables cuantitativas se efectuó mediante prueba de *t* de Student o de U-Mann-Whitney, según si cumplían o no hipótesis de normalidad, respectivamente. El nivel de significación estadística se estableció en *p* < 0,05. El análisis estadístico se realizó empleando SPSS v29 (IBM, Armonk, NY, USA).

## 3. Resultados

Entre enero de 2017 y agosto de 2023, se incluyeron 40 pacientes con el criterio de inclusión de ingreso hospitalario con diagnóstico principal de enfermedad neuroinvasiva por virus del Nilo Occidental. En cuanto a las características demográficas, la mediana de edad fue de 65 años (rango intercuartílico (RIQ) de 37–77 años) y el porcentaje de mujeres fue del 45%. Eran fumadores un 25%, 1 persona padecía alcoholismo y ninguna presentaba consumo de otras drogas. 45% eran hipertensos, 25% dislipémicos, 23% diabéticos, 13% habían padecido un ictus, 10% cardiopatía isquémica, 8% enfermedad pulmonar obstructiva crónica, 8% enfermedad renal crónica, 8% estaban inmunodeprimidos, 5% insuficiencia cardiaca, 2 personas obesas y 1 persona con claudicación intermitente. El 95% tenía buena situación basal, con mRs ≤2.

Respecto a los síntomas, el 95% de los pacientes presentó fiebre y el 85% refirió síntomas prodrómicos previos a la aparición de manifestaciones neurológicas, como se detalla en la Fig. 1. Se documentaron síntomas prodrómicos en 34 de los 40 pacientes. Excluyendo la fiebre y la cefalea (analizadas por separado), los más frecuentes fueron astenia o malestar general, náuseas, vómitos y artralgias. En cuanto a los síntomas neurológicos, los más comunes fueron alteración de conciencia (73%), síndrome confusional (60%), cefalea (50%), ataxia (33%), alteración del lenguaje (33%) y rigidez nucal (33%). 33% presentaron mialgias, 15% presentaron exantema cutáneo y 10% otalgia. 24 (60%) fueron diagnosticados de encefalitis, 10 (25%) de meningitis y 6 (15%) de parálisis flácida aguda, según criterios clínicos. Se registraron crisis epilépticas en 8 pacientes, todas diagnosticadas mediante evaluación clínica, ya fuera por observación directa durante la hospitalización o a través de la anamnesis. No se detectaron crisis electrográficas en los EEG realizados y no se documentaron casos de estatus epiléptico. La mediana de días de hospitalización fue de 10 días (RIQ 5–17). 48% requirió oxigenoterapia, 25% ventilación mecánica y 13% instauración de aminas. Requirieron ingreso en UCI el 33%. 13% padecieron shock (definido como hipotensión arterial refractaria a medidas de reposición, incluyendo aminas). 45% presentaron complicaciones sistémicas. 15% de los pacientes fallecieron durante el ingreso.

**Fig. 1.** **Descripción de sintomatología neurológica, ordenada por frecuencia de aparición**.

En cuanto al estudio diagnóstico, se realizó determinación de serología y PCR de VNO en orina en todos los casos, así como PCR de VNO en LCR en 98%. El perfil serológico al diagnóstico consistió en 65% con IgM+ e IgG–, 30% IgM+ e IgG+, 1 paciente con IgM– e IgG+ y 1 paciente con IgM– e IgG–. 13% obtuvo resultado positivo en PCR de VNO en orina. También 13% obtuvo resultado similar en PCR de VNO en LCR. 19 casos (48%) cumplían criterios de caso confirmado y 21 (53%) fueron clasificados como casos probables (**Tabla 2 del Material Suplementario**). Sobre el estudio del LCR, los resultados de citobioquímica se muestran en la Tabla 1, donde destacamos que las medianas de proteínas totales (62 mg/dL) y leucocitos (55 células/mm^3^) se encontraban en rango patológico (>50 mg/dL y >5 células/mm^3^, respectivamente), con claro predominio monocítico (mediana de 35% de polimorfonucleares). De los 39 pacientes con estudio citológico de LCR disponible, 17 (38%) presentaron una predominancia de polimorfonucleares (>50% del total de leucocitos). Este patrón se observó en distintos síndromes clínicos, incluyendo encefalitis y meningitis. 95% de las punciones lumbares resultaron atraumáticas. 90% de los LCR presentaron aspecto claro. Sólo 5% presentó consumo de glucosa. Las determinaciones cuantitativas emparejadas de inmunoglobulinas en LCR y suero no estaban disponibles de forma sistemática en nuestra cohorte, por lo que no fue posible realizar un análisis completo mediante el diagrama de Reiber.

**Tabla 1.** **Perfil global de resultados de citobioquímica de LCR (n = 39)**.

Citobioquímica de LCR	Mediana	RIQ	P25	P75
Glucosa (mg/dL)	68	32	58	90
Proteínas totales (mg/dL)	62	50	43	93
Leucocitos (células/mm^3^)	55	146	17	163
Polimorfonucleares (% sobre total de leucocitos)	35	52	16	68
Hematíes (células/mm^3^)	0	3	0	3

LCR, líquido cefalorraquídeo; RIQ, rango intercuartílico; P25, percentil 25; P75, percentil 75.

Sobre los estudios de neuroimagen, todos disponían de TC craneal urgente, resultando normal en 98%. Se obtuvo RM craneal durante el ingreso en el 60%, siendo patológica en 25% respecto al total muestral, con hallazgo más frecuente de hiperintensidad en secuencia T2 en troncoencéfalo y tálamo. Los pacientes con hallazgos patológicos en la RM cerebral presentaron puntuaciones más altas en la escala mRS tanto a los 12 como a los 24 meses de seguimiento (medianas de 4,5 y 5, respectivamente) en comparación con aquellos con neuroimagen normal (mediana de 2 en ambos casos). No obstante, estas diferencias no fueron estadísticamente significativas (*p* = 0,202 y *p* = 0,312, respectivamente; prueba de Mann–Whitney). Se realizó EEG en 48%, siendo el resultado más frecuente normal (18%), seguido de disfunción leve generalizada (13%) y moderada (10%), respecto al total de la muestra. Solo un paciente presentó descargas epileptiformes en el EEG, sin crisis electrográfica ni criterios de estatus epiléptico no convulsivo.

Sobre el tratamiento, 63% recibió antivirales (fundamentalmente aciclovir), 65% antibióticos y 33% anticomiciales (8 pacientes los recibieron con intención terapéutica, tras la aparición de crisis clínicas; los 5 restantes fueron tratados de forma profiláctica, principalmente en el contexto de ingreso en UCI, ante la falta de monitorización EEG continua, y siguiendo el criterio clínico de los intensivistas para evitar dejar crisis subclínicas sin tratar). El aciclovir se inició en todos los pacientes en el momento de la sospecha de infección del sistema nervioso central, y en general se mantuvo hasta poder descartar razonablemente una encefalitis herpética. En la mayoría de los casos, esto coincidió con la realización de una segunda punción lumbar negativa o la confirmación de un diagnóstico alternativo (virus del Nilo Occidental). Aunque no se dispuso de la duración exacta del tratamiento en cada paciente, la mayoría recibió un ciclo completo o una parte sustancial del mismo. 30% no recibió inmunomoduladores; de los que sí, 53% únicamente corticosteroides y 18% tanto corticosteroides como inmunoglobulinas.

Junto al estudio de la muestra en su conjunto, se analizó con especial atención a los pacientes según si requirieron ingreso o no en UCI. Se apreció que en aquellos que requirieron ingreso en UCI, los tres síntomas neurológicos más frecuentes eran alteración de conciencia (92%), síndrome confusional (61%) y crisis comicial (46%), este último con diferencia estadísticamente significativa respecto a los que no precisaron ingreso en UCI (46% vs 7%, *p *= 0,008), como se muestra en Fig. 2.

**Fig. 2.** **Descripción de síntomas neurológicos según precisaron o no ingreso en Unidad de Cuidados Intensivos (UCI) (n = 40)**. *p < 0,05.

Respecto a las características demográficas, antecedentes personales y otros síntomas, no hubo diferencias estadísticamente significativas en la mayoría de parámetros entre grupos, salvo por mayor número de días de hospitalización en los que requirieron ingreso en UCI (17 vs 7; *p* < 0,001). En cuanto a la citobioquímica de LCR, tampoco hubo diferencias estadísticamente significativas. Sí se encontraron diferencias en cuanto a mayor presencia de shock (31% vs 4%, *p* = 0,031), necesidad de oxigenoterapia (92% vs 26%; *p* < 0,001), ventilación mecánica (62% vs 7%; *p* < 0,001), uso de aminas (31% vs 4%; *p* = 0,031), complicaciones sistémicas (77% vs 30%; *p* = 0,007), menor uso de corticosteroides de forma aislada (46% vs 56%; *p* = 0,002) y su mayor uso de forma combinada con inmunoglobulinas (46% vs 4%; *p* = 0,002). No hubo diferencias estadísticamente significativas en el resto de variables, incluyendo neuroimagen, EEG, situación funcional a lo largo del seguimiento ni perfil diagnóstico para el VNO en las distintas muestras analizadas.

Respecto a la situación funcional, se muestran resultados en Fig. 3. El 95% presentaba una buena situación basal. Sin embargo, al alta (46%), a los 12 (48%) y 24 meses (48%) se produce un decremento de más del 40% de pacientes con buena situación funcional respecto al nivel basal. A su vez, se observa un cambio significativo en cuanto al empeoramiento de la situación funcional (incremento ≥2 en mRS al comparar situación basal y a los 24 meses) en 42,5% de los pacientes. Se observó una correlación positiva moderada y estadísticamente significativa entre la edad y el grado de discapacidad funcional tanto a los 12 meses (rho de Spearman = 0,475; *p* = 0,002) como a los 24 meses (rho = 0,549; *p* < 0,001), lo que sugiere que una mayor edad se asoció con peores resultados a largo plazo. La fuerza de la asociación fue ligeramente superior a los 24 meses. Los intervalos de confianza al 95% fueron 0,182–0,690 y 0,277–0,739, respectivamente. La mediana de seguimiento fue 35 meses (RIQ 35–36), concentrándose la mayoría de casos en verano de 2020, principalmente en zonas de La Puebla y Coria del Río.

**Fig. 3.** **Situación funcional a lo largo del tiempo de seguimiento (n = 40), mostrándose según puntuación en escala de Rankin modificada (mRS) (0–6)**.

No encontramos diferencias estadísticamente significativas entre el hallazgo de LCR patológico (definido como aumento de proteínas y/o células por encima de valores de normalidad según laboratorio local de referencia) o el tratamiento respecto a la situación funcional en los distintos momentos del seguimiento; tampoco respecto al subtipo clínico que presentaron los pacientes y la mortalidad registrada. Al comparar a los pacientes según su desenlace funcional a largo plazo (mRS ≤2 vs >2), no se encontraron asociaciones estadísticamente significativas con las características basales, factores de riesgo vascular, hallazgos del LCR, tipo de presentación clínica ni tratamientos administrados. Estos resultados probablemente reflejan la escasa potencia estadística del estudio y deben interpretarse con precaución.

## 4. Discusión

En el presente estudio, reportamos 40 casos de pacientes con enfermedad neuroinvasiva por VNO en un contexto regional endémico. Los hallazgos refuerzan la considerable repercusión clínica y funcional de esta patología, caracterizada por una elevada mortalidad (15% intrahospitalaria y 23% a los 24 meses) y un deterioro funcional significativo en casi la mitad de los pacientes tras dos años de seguimiento.

En cuanto a las características demográficas, la edad mediana de 65 años observada en nuestra cohorte coincide con lo descrito en otras series sobre la enfermedad neuroinvasiva por virus del Nilo Occidental, que afecta predominantemente a adultos mayores [9, 10, 11]. El 8% de los pacientes estaban inmunodeprimidos, una condición que ha sido asociada a un peor pronóstico vital en estudios previos [17]; sin embargo, en nuestra muestra no se evidenció una relación clara con una evolución más desfavorable. La encefalitis fue la manifestación clínica más frecuente (73%), seguida de meningitis (33%) y parálisis flácida aguda (25%), con cierto grado de solapamiento clínico entre ellas. Aunque no se observaron diferencias significativas en las características basales, la mortalidad o la evolución funcional a largo plazo entre los distintos subtipos clínicos, estas comparaciones deben interpretarse con precaución debido a la limitada potencia estadística del estudio. No obstante, la distribución de los síndromes clínicos y sus desenlaces es coherente con lo descrito en otras series, reforzando el papel de la encefalitis como la forma principal de afectación neuroinvasiva por VNO. Este patrón clínico coincide con estudios previos que destacan la asociación entre encefalitis, inflamación endotelial y disrupción de la barrera hematoencefálica, mecanismos bien descritos en las infecciones neuroinvasivas por flavivirus como el virus del Nilo Occidental. Aunque en nuestra cohorte no se documentaron eventos cerebrovasculares, hemos mantenido esta discusión debido a la creciente evidencia que sugiere que la encefalitis viral puede inducir daño endotelial vascular y un entorno proinflamatorio y protrombótico, lo cual podría predisponer a complicaciones vasculares, como el ictus, incluso más allá de la fase aguda. Estos procesos, respaldados por datos clínicos y experimentales, no son exclusivos del VNO, sino que se han observado en otros flavivirus neurotrópicos. Nuestro objetivo no es extrapolar más allá de los datos obtenidos, sino contextualizar nuestros hallazgos, especialmente la alta frecuencia de encefalitis, en un marco fisiopatológico más amplio. Esta perspectiva es coherente con discusiones emergentes en la literatura y tiene un carácter exploratorio e hipotético, que subraya la necesidad de futuras investigaciones sobre las secuelas cerebrovasculares en pacientes con enfermedad neuroinvasiva por VNO [7, 12, 13].

En comparación con lo publicado, nuestros datos son similares a una serie de menor tamaño en la misma provincia, donde se encontró un 63% de casos de encefalitis [16]. Sin embargo, difieren de otras regiones donde la meningitis fue la manifestación más frecuente [9, 11]. Además, el 33% de nuestra muestra requirió ingreso en UCI, y el 25% necesitó ventilación mecánica. La mortalidad en nuestra serie fue superior a la reportada en otras publicaciones, con un 15% intrahospitalaria y un 23% a los 24 meses, frente al 10% en estudios previos [9, 10]. No encontramos diferencias en la mortalidad según las manifestaciones clínicas, hecho también conocido en la literatura [9].

El diagnóstico de la enfermedad neuroinvasiva por virus del Nilo Occidental requiere un enfoque multimodal que combine la evaluación clínica con herramientas serológicas y moleculares. En nuestra cohorte, el diagnóstico se basó principalmente en la detección de IgM e IgG en suero y LCR, así como en la PCR, realizada sobre todo en LCR u orina. Aunque la IgM en LCR se considera un criterio confirmatorio, su persistencia en el tiempo puede generar ambigüedad diagnóstica, y la IgM en suero es susceptible a falsos positivos por reactividad cruzada con otros flavivirus o por antecedentes vacunales [9, 10, 11]. La PCR, si bien menos frecuentemente positiva, resultó diagnóstica en el 13% de nuestros pacientes, y tiene especial utilidad en las fases precoces de la infección o en pacientes inmunodeprimidos [10]. Estos hallazgos refuerzan la importancia de combinar diferentes métodos diagnósticos para mejorar la precisión y minimizar el riesgo de errores de clasificación [9, 10, 11].

En cuanto al estudio complementario, el LCR presentó un perfil inflamatorio con predominio linfocítico, elevación de proteínas y ausencia de consumo de glucosa en la mayoría de los casos, consistente con lo esperado. La predominancia de neutrófilos en el LCR, observada en el 38% de nuestros casos, ha sido descrita previamente en la enfermedad neuroinvasiva por virus del Nilo Occidental, así como en otras infecciones por flavivirus como la encefalitis por virus transmitido por garrapatas. Aunque este hallazgo es poco frecuente en meningitis o encefalitis de origen viral, puede representar una pista diagnóstica precoz que oriente hacia WNND, especialmente en contextos epidemiológicos compatibles (Pelz *et al*., 2024 [18]; Senel *et al*., 2020 [19]). Nuestros resultados respaldan su consideración dentro del razonamiento diagnóstico diferencial en infecciones del Sistema Nervioso Central (SNC) en áreas endémicas. Aunque se ha descrito la utilidad del diagrama de Reiber para detectar síntesis intratecal precoz de IgM en casos de enfermedad neuroinvasiva por virus del Nilo Occidental [18, 19], en nuestra cohorte no fue posible realizar este análisis, ya que no se disponía de determinaciones cuantitativas emparejadas de inmunoglobulinas en LCR y suero de forma sistemática. Respecto a la neuroimagen, aunque los hallazgos en otras series varían, en nuestra muestra prevaleció la afectación diencefálica (principalmente talámica) y troncoencefálica. Aunque las diferencias en la situación funcional no alcanzaron significación estadística, se observa una tendencia clínica relevante: los pacientes con hallazgos patológicos en la resonancia, en particular lesiones en estructuras profundas como el tálamo o el tronco encefálico, mostraron peor recuperación a largo plazo. Este patrón ha sido descrito en otras series y sugiere que el compromiso estructural del SNC podría asociarse a una mayor discapacidad persistente [9, 10, 11, 16, 17].

El impacto funcional de la enfermedad neuroinvasiva por virus del Nilo Occidental es particularmente relevante, ya que casi la mitad de los pacientes de nuestra cohorte experimentaron un deterioro significativo en su calidad de vida, pasando de una independencia total a requerir ayuda para las actividades de la vida diaria o incluso cuidados continuos. Esta observación es coherente con estudios previos que reportan tasas de recuperación completa inferiores al 40% al año de seguimiento [9, 10]. En nuestra cohorte, el deterioro funcional persistió hasta los 24 meses, con una mediana de seguimiento superior a dos años y medio, lo que refuerza la necesidad de considerar la infección como una enfermedad con potencial de discapacidad crónica. Además, nuestros datos sugieren que la edad avanzada se asocia a una peor recuperación funcional a largo plazo, una tendencia que también ha sido documentada en investigaciones previas sobre enfermedades neuroinfecciosas, incluida la infección por VNO.

Aunque las trayectorias de recuperación pueden variar, solo una parte de los pacientes alcanza una recuperación funcional completa, y muchos continúan presentando déficits neurológicos persistentes durante meses o incluso años tras la enfermedad aguda. La cohorte de Houston sobre virus del Nilo Occidental constituye una de las evidencias prospectivas más sólidas sobre este tema, demostrando que el deterioro neurológico persistía en casi la mitad de los pacientes un año después de la infección, especialmente en aquellos con encefalitis o parálisis flácida aguda. En particular, la parálisis flácida aguda se ha asociado de forma consistente con los peores desenlaces a largo plazo, debido a la lesión irreversible de las neuronas del asta anterior y la pérdida de motoneuronas. Del mismo modo, la encefalitis suele dejar secuelas cognitivas y motoras residuales, mientras que la meningitis se asocia generalmente a una recuperación más favorable, aunque pueden persistir síntomas como fatiga o alteraciones neurocognitivas sutiles [20]. Estos hallazgos refuerzan la necesidad de una rehabilitación neurológica precoz, un seguimiento estructurado a largo plazo, y una mayor conciencia clínica sobre el impacto crónico que la Enfermedad Neuroinvasiva del Nilo Occidental (ENNO) puede tener en los supervivientes.

El manejo terapéutico sigue siendo principalmente de soporte, ya que no se ha demostrado que los antivirales o inmunomoduladores utilizados tengan un impacto significativo en el pronóstico a largo plazo [11]. Los pacientes que requirieron ingreso en UCI presentaron mayor prevalencia del fenotipo encefalítico (92%), estancias hospitalarias más prolongadas, mayor gravedad sistémica y mayor necesidad de soporte invasivo. Sin embargo, no encontramos asociación entre la evolución funcional y la citobioquímica del LCR, lo que no permite respaldar su utilidad como factor pronóstico.

Estos hallazgos subrayan la importancia de implementar estrategias preventivas más efectivas, como el control vectorial y la vigilancia epidemiológica, especialmente frente al impacto del cambio climático en la expansión de enfermedades transmitidas por vectores [4]. Además, el deterioro funcional observado refuerza la necesidad de destinar recursos a la rehabilitación y al cuidado de pacientes dependientes, considerando que casi el 50% de los pacientes no recuperaron su funcionalidad basal tras 24 meses de seguimiento [5, 11].

Finalmente, el vínculo entre el cambio climático, el daño endotelial y el aumento de la carga de ictus asociado a infecciones virales como el VNO debería ser un foco clave de investigación futura [7, 12, 13]. Comprender estos mecanismos permitiría desarrollar intervenciones integrales que reduzcan el impacto a largo plazo de estas enfermedades. Como limitaciones, cabe destacar la naturaleza retrospectiva del presente estudio y el reducido tamaño muestral en los análisis por subgrupos, lo que podría limitar la capacidad de identificar diferencias estadísticamente significativas.

## 5. Conclusiones

La enfermedad neuroinvasiva por VNO, pese a ser infrecuente, tiene un impacto considerable en la situación funcional de los pacientes, así como elevada mortalidad. Resulta imprescindible enfocar más recursos en medidas preventivas para evitar brotes epidémicos dada la falta de eficacia demostrada del tratamiento actual del tratamiento actual y la posibilidad de persistencia de otros factores mórbidos que expliquen el incremento de la incapacidad y mortalidad diferida en el tiempo. Junto a la prevención primaria como pilar básico, el destino de recursos a rehabilitación y atención a la dependencia es fundamental en aquellos que sufren un mayor deterioro de su situación funcional, por su gran repercusión.

## Data Availability

The datasets used and analyzed during the current study are available from the corresponding author on reasonable request.

## Supplementary Material

Supplementary material associated with this article can be found, in the online version, at https://doi.org/10.31083/RN36787.

## References

[1] Magallanes S, Llorente F, Ruiz-López MJ, Puente JMDL, Ferraguti M, Gutiérrez-López R (2024). Warm winters are associated to more intense West Nile virus circulation in southern Spain. *Emerging Microbes & Infections*.

[2] García San Miguel Rodríguez-Alarcón L, Fernández-Martínez B, Sierra Moros MJ, Vázquez A, Julián Pachés P, García Villacieros E (2021). Unprecedented increase of West Nile virus neuroinvasive disease, Spain, summer 2020. *Euro Surveillance: Bulletin Europeen Sur Les Maladies Transmissibles = European Communicable Disease Bulletin*.

[3] García-Carrasco JM, Muñoz AR, Olivero J, Segura M, Real R (2021). Predicting the spatio-temporal spread of West Nile virus in Europe. *PLoS Neglected Tropical Diseases*.

[4] Aguilera-Sepúlveda P, Cano-Gómez C, Villalba R, Borges V, Agüero M, Bravo-Barriga D (2024). The key role of Spain in the traffic of West Nile virus lineage 1 strains between Europe and Africa. *Infectious Diseases (London, England)*.

[5] de Freitas Costa E, Streng K, Avelino de Souza Santos M, Counotte MJ (2024). The effect of temperature on the boundary conditions of West Nile virus circulation in Europe. *PLoS Neglected Tropical Diseases*.

[6] Lu L, Zhang F, Oude Munnink BB, Munger E, Sikkema RS, Pappa S (2024). West Nile virus spread in Europe: Phylogeographic pattern analysis and key drivers. *PLoS Pathogens*.

[7] Erazo D, Grant L, Ghisbain G, Marini G, Colón-González FJ, Wint W (2024). Contribution of climate change to the spatial expansion of West Nile virus in Europe. *Nature Communications*.

[8] Ndione MHD, Ndiaye EH, Faye M, Diagne MM, Diallo D, Diallo A (2022). Re-Introduction of West Nile Virus Lineage 1 in Senegal from Europe and Subsequent Circulation in Human and Mosquito Populations between 2012 and 2021. *Viruses*.

[9] Debiasi RL, Tyler KL (2006). West Nile virus meningoencephalitis. *Nature Clinical Practice. Neurology*.

[10] Petersen LR, Brault AC, Nasci RS (2013). West Nile virus: review of the literature. *JAMA*.

[11] Sejvar JJ, Haddad MB, Tierney BC, Campbell GL, Marfin AA, Van Gerpen JA (2003). Neurologic manifestations and outcome of West Nile virus infection [published correction in JAMA. 2003; 290: 1318. https://doi.org/10.1001/jama.290.10.1318-b]. *JAMA*.

[12] Burakoff A, Lehman J, Fischer M, Staples JE, Lindsey NP (2018). West Nile Virus and Other Nationally Notifiable Arboviral Diseases - United States, 2016. *MMWR. Morbidity and Mortality Weekly Report*.

[13] Clarke M, Falcione S, Boghozian R, Todoran R, Zhang Y, C Real MG (2024). Viral Infection and Ischemic Stroke: Emerging Trends and Mechanistic Insights. *Journal of the American Heart Association*.

[14] Moreno-Reina C, Martínez-Moya M, Piñero-González de la Peña P, Caro-Domínguez P (2022). Neuroinvasive disease due to West Nile virus: Clinical and imaging findings associated with a re-emerging pathogen. *Radiologia*.

[15] Cuenca-Apolo DX, García-Delgado Rosado H, Amaya Villar R (2022). West Nile virus encephalitis outbreak in the Guadalquivir river basin area: experience and results in patients admitted to the intensive care unit. *Medicina Intensiva*.

[16] Hiraldo JDG, Fuerte-Hortigón A, Domínguez-Mayoral A, De la Rosa Riestra S, Palacios-Baena ZR, Fernández FS (2023). Uncovering the neurological effects of West Nile virus during a record-breaking southern Spain outbreak in 2020-2021. *Journal of Neuroimmunology*.

[17] Mbonde AA, Gritsch D, Harahsheh EY, Kasule SN, Hasan S, Parsons AM (2024). Neuroinvasive West Nile Virus Infection in Immunosuppressed and Immunocompetent Adults. *JAMA Network Open*.

[18] Pelz JO, Mühlberg C, Friedrich I, Weidhase L, Zimmermann S, Maier M (2024). A Specific Pattern of Routine Cerebrospinal Fluid Parameters Might Help to Identify Cases of West Nile Virus Neuroinvasive Disease. *Viruses*.

[19] Senel M, Rapp D, Mayer B, Jesse S, Süssmuth SD, Otto M (2020). Tick-Borne Encephalitis: A Differential Pattern of Intrathecal Humoral Immune Response and Inflammatory Cell Composition Compared with Other Viral CNS Infections. *Cells*.

[20] Weatherhead JE, Miller VE, Garcia MN, Hasbun R, Salazar L, Dimachkie MM (2015). Long-term neurological outcomes in West Nile virus-infected patients: an observational study. *The American Journal of Tropical Medicine and Hygiene*.
